# Supplementary material for: Capturing chemical reactions inside biomolecular condensates with reactive Martini simulations
Source: Commun Chem. 2024 Jul 4;7:151. doi: 10.1038/s42004-024-01234-y (PMC11222477; doi:10.1038/s42004-024-01234-y)
Supplement: Supplementary file 2 — Supplementary Information [file 42004_2024_1234_MOESM2_ESM.pdf]

Supplementary information for: Capturing chemical reactions inside biomolecular condensates with reactive Martini simulations

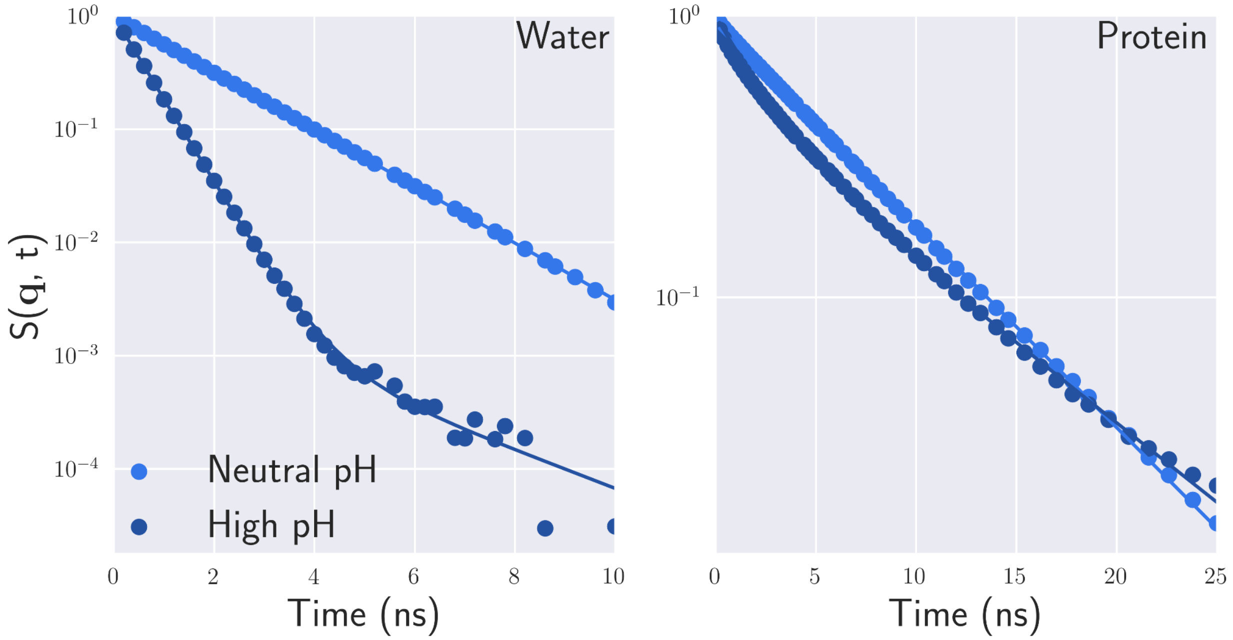

Figure S1: Incoherent scattering function data, and fitted curves in the LFssFL peptide systems for water (left) and peptide (right) components. The top row shows the systems at neutral pH, and the bottom row at high pH, with N termini deprotonated. The fitted results are summarised in table S1 below, and the fitting statistics for single and double exponential models in table S2.

	Water ($x10^{-9}$ m^2s^{-1})		Peptide ($x10^{-12}$ m^2s^{-1})	
	D_{Bulk}	$D_{Condensate}$	D_{Bulk}	$D_{Condensate}$
Neutral pH	1.85 ± 0.01	-	465 ± 1	-
High pH	2.07 ± 0.01	0.34 ± 0.03	324 ± 7	76 ± 1

Table S1. Diffusion coefficients for the water and peptide components of the LFssFL/water system at neutral and high pH, as measured from the fits to the incoherent scattering functions in the Figure S1.

	Water		Peptide	
	Single exponential model	Double exponential model	Single exponential model	Double exponential model
Neutral pH	-864.33	-862.36	-977.29	-896.01
High pH	-741.5	-827.71	-728.6	-819.29

Table S2. Akaike information criterion values for single and double exponential model fits to the incoherent scattering function data shown in figure S1.

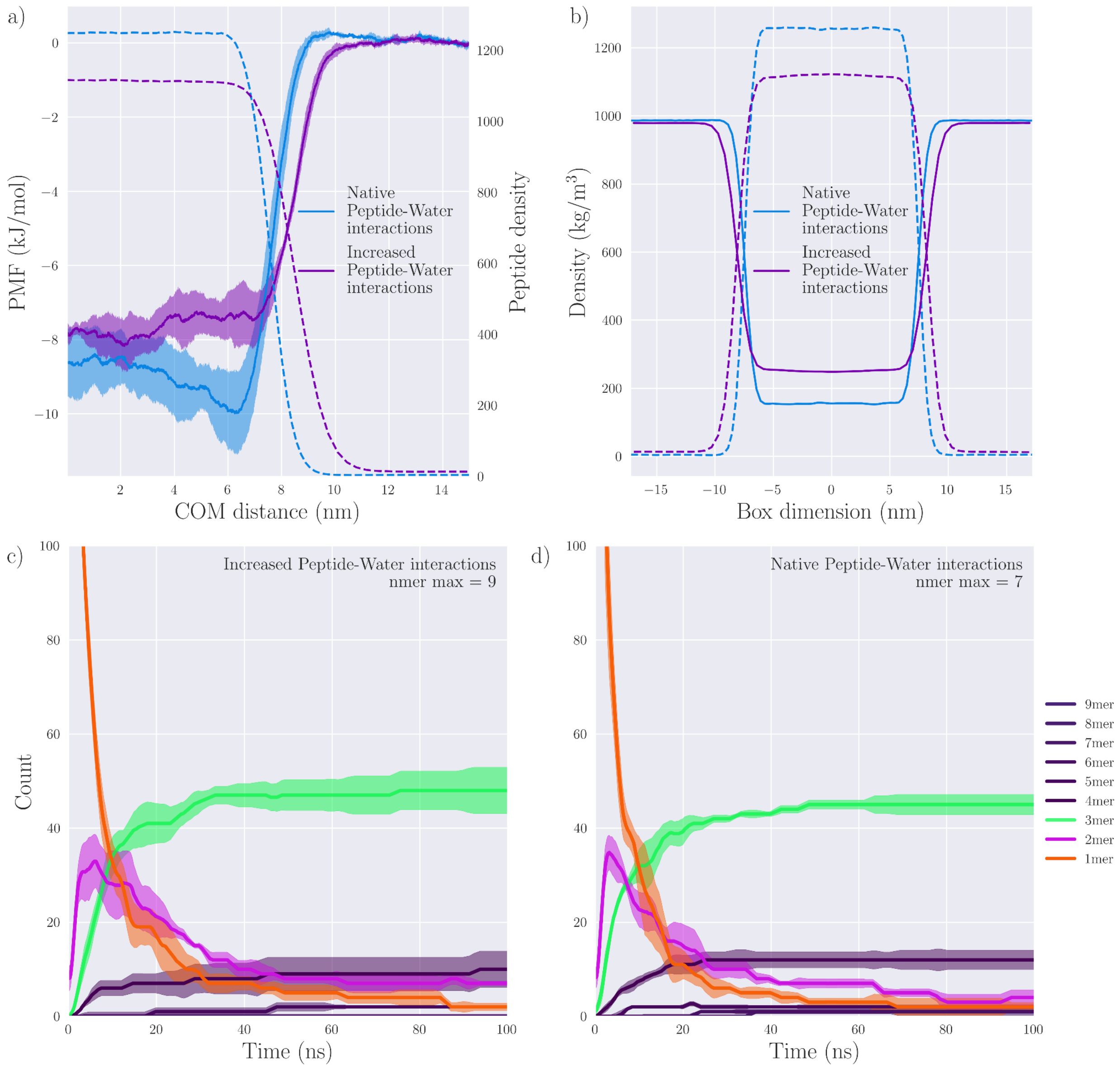

Figure S2. Comparing systems with increased peptide-water interactions. Data for native systems are taken from the main text and replotted here for clarity of comparison. a) PMF of a benzene-1,3-diol molecule moving from the dense to dilute region of a condensate at high pH, comparing the native Martini model of LFssFL (blue) with a model with slightly stronger peptide-water interactions (purple). b) density profiles of the condensates of the two models showing the protein (dashed lines) and water (solid lines) components of the two systems. The weight proportion of water inside the condensate has been increased from around 17% to around 22%. c,d) ring size changes between the two condensate systems with native and stronger peptide-water interactions.

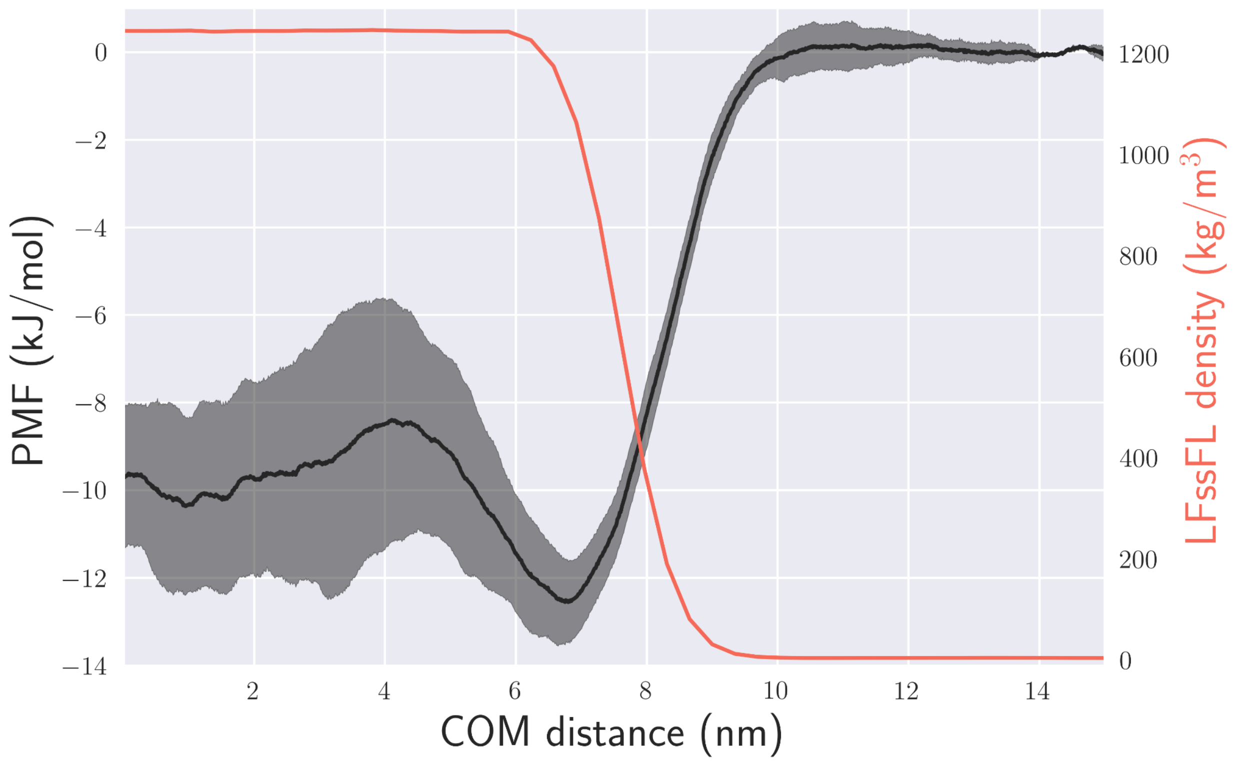

Figure S3. Potential of mean force of a molecule of XGLKFK across the interface of a LFssFL condensate.

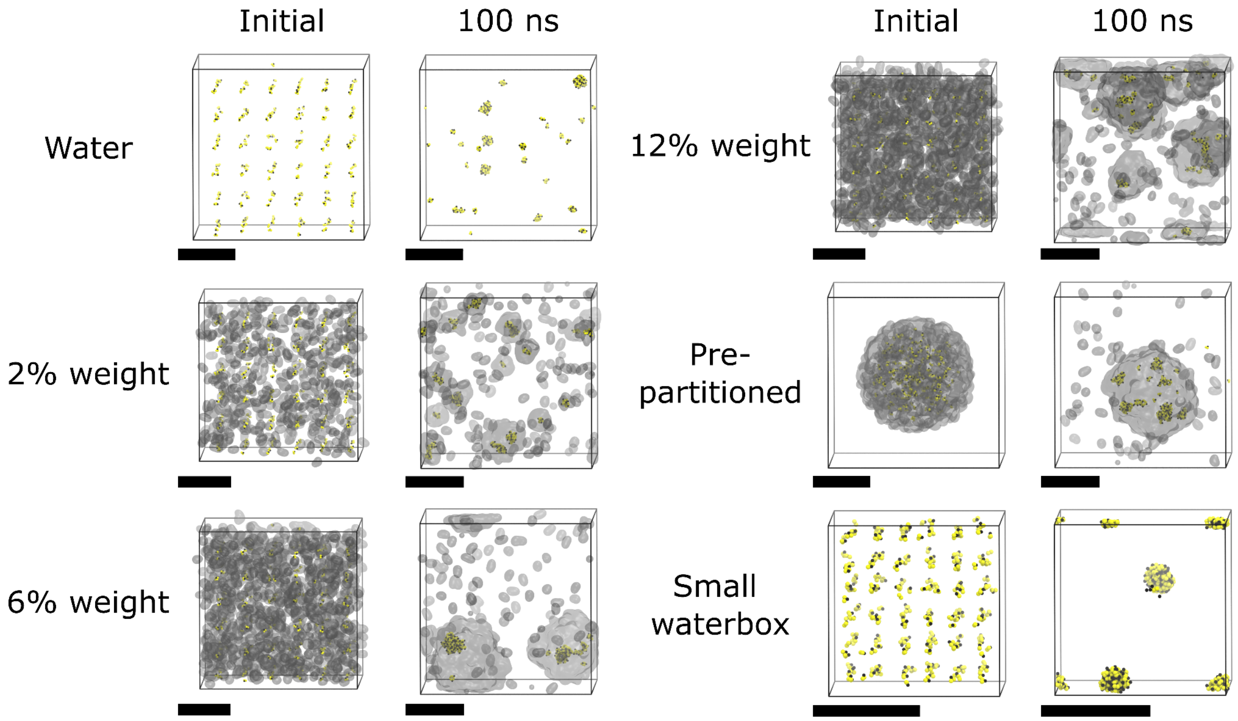

Figure S4: initial and final configurations of simulations as described in the main text. Water removed for clarity. Peptides shown as transparent surfaces, and the reactive molecules shown in yellow and black. All scale bars are 100 A.

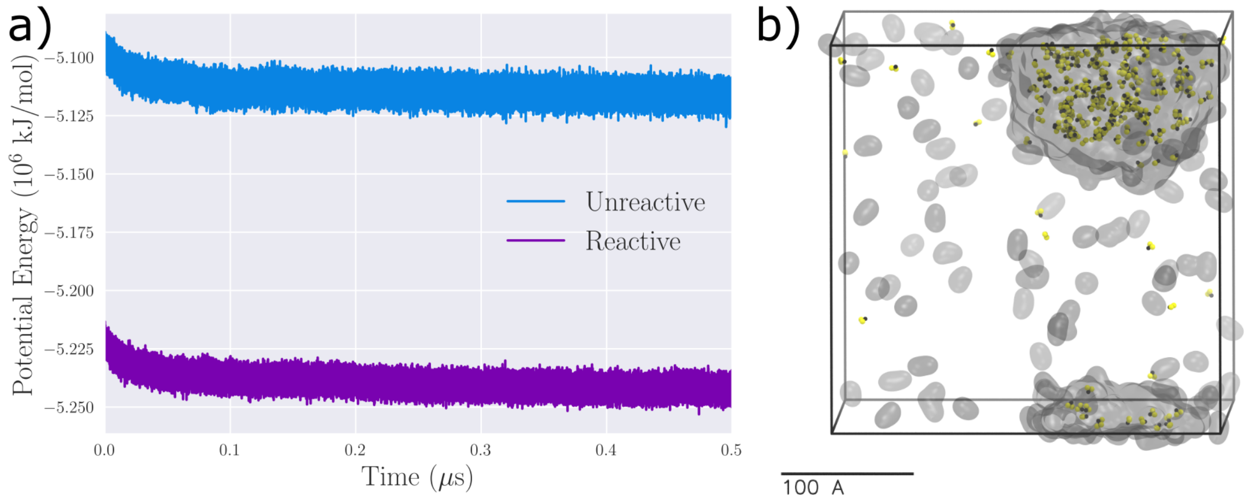

Figure S5: Reactions of benzene-1,3-dithiol do not affect condensate formation. a) System potential energy for unreactive and reactive systems. b) Simulation snapshot of LFssFL peptides having formed a condensate with unreactive benzene-1,3-dithiol molecules

**System conditions**	Water	2% weight	6% weight	12% weight	Pre-partitioned	Small waterbox
Number of peptides	0	500	1500	2500	1500	0
Number of waters	220760	211252	220760	192912	199895	31414
Number of reactive molecules	216	216	216	216	216	216
Peptide weight proportion	n/a	2%	6%	12%	6%	n/a
Cubic box length (nm)	30	30	30	30	30	16

Table S3. The initial prepared conditions for the reactive simulations performed in this work.

System	1mer	2mer	3mer	4mer	5mer	6mer	7mer	8mer	9mer	10 mer	11 mer	12 mer	13 mer	14 mer	15 mer
Pre-partitioned (Condensate phase)	0	3	1	26	7	6	4	1	0	0	1	0	1	1	1
Water (Dilute phase)	0	2	3	48	14	1	1	0	0	0	0	0	0	0	0
Small waterbox (Dilute phase)	0	0	1	28	11	6	5	1	1	1	1	0	0	0	0
2% weight (Condensate phase)	0	3	5	42	13	2	1	0	0	0	0	0	0	0	0
6% weight (Condensate phase)	0	0	0	45	13	2	3	1	0	0	0	0	0	0	0
12% weight (Condensate phase)	0	2	6	46	13	1	1	1	0	0	0	0	0	0	0

Table S4. Final ring counts for each system.

Reaction rate (molecules/s/m^3)	Pre-partitioned	Water	Small waterbox	2% weight	6% weight	12% weight
Dilute phase	n/a	2.48e-04	1.90e-03	n/a	n/a	n/a
Dense phase (inside condensate)	1.69e-03	n/a	n/a	n/a	n/a	n/a
Co-formation	n/a	n/a	n/a	2.55e-04	2.59e-04	2.56e-04

Table S5. Reaction rates for each system, denoted by the region of the system the reaction is taking place in. Note for co-formation simulations, the dense and dilute regions are not well defined, and it is not possible to distinguish where a reaction specifically occurs.

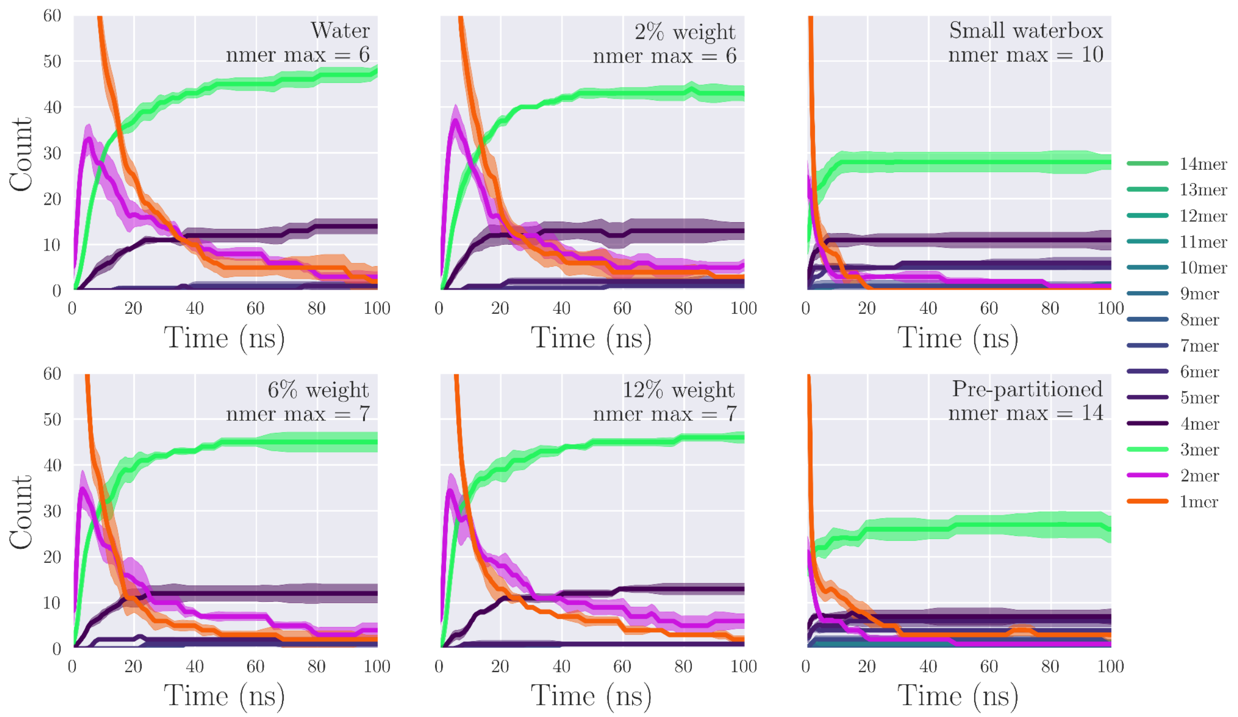

Figure S6: Change in ring sizes across each simulation, median taken from 3 repeats.

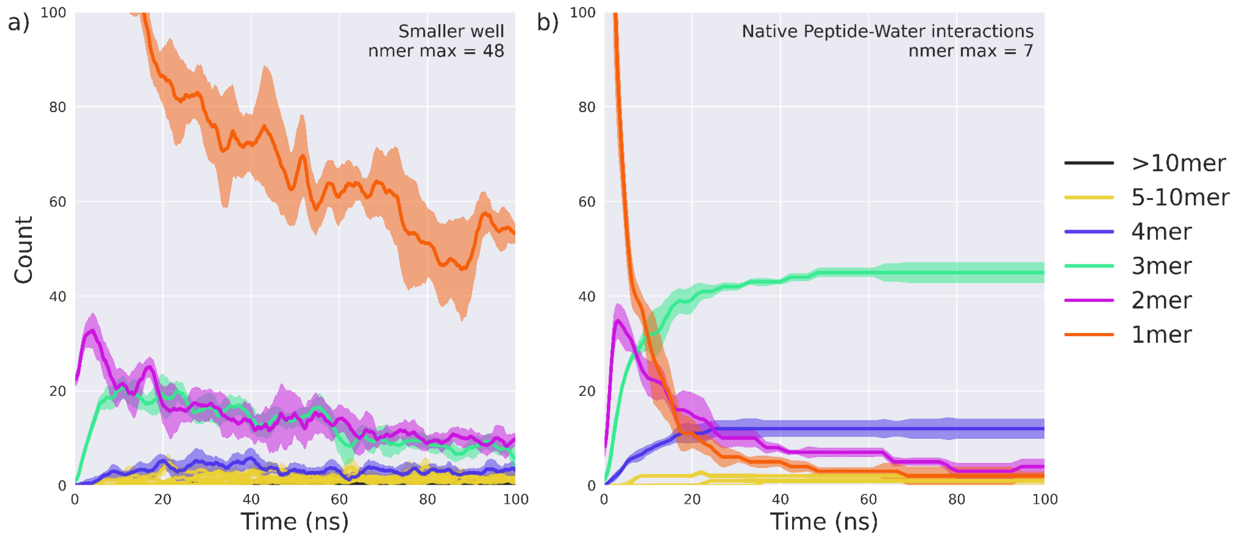

Figure S7: Ring size counts using smaller reactive potential well. a) Ring counts for reactive Martini simulations with a potential well of 20 kJ/mol, as compared to the default 60 kJ/mol potential. b) Ring size counts in the native potential systems taken from the main text and replotted here for clarity of comparison.

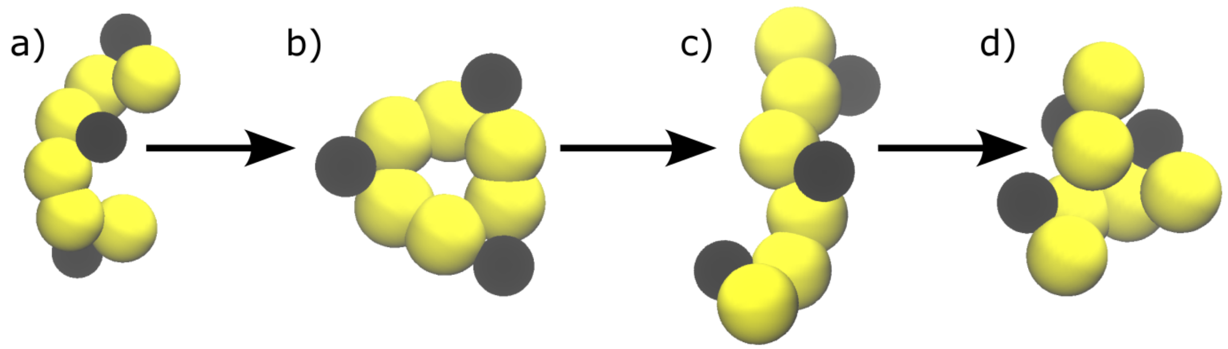

Figure S8. Illustration of reversible bond formation using a smaller reactive potential. a) Three monomers come together to form a strand. b) The strand self-completes and forms a ring. c) The ring breaks apart again. d) One monomer breaks away from the strand.

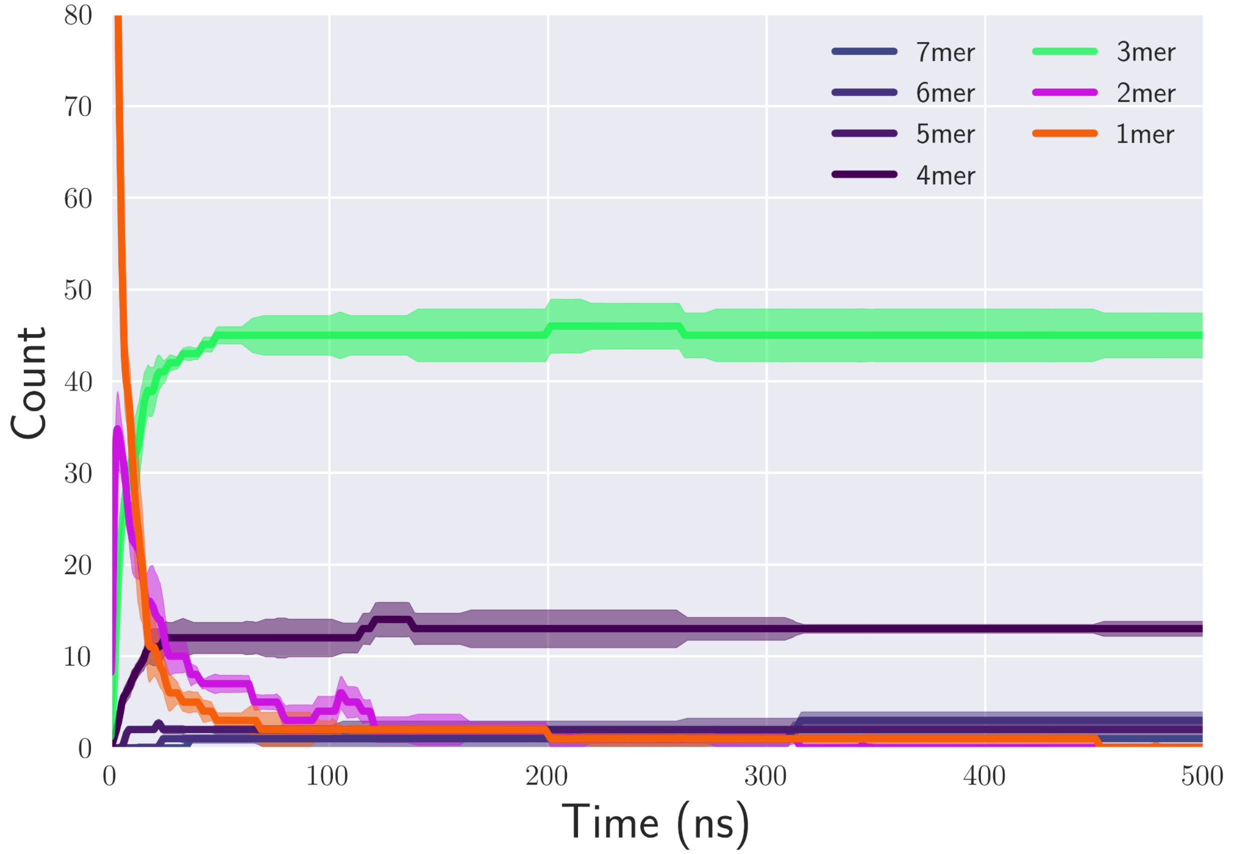

Figure S9. Ring size counts over a longer simulation trajectory of 500 ns.

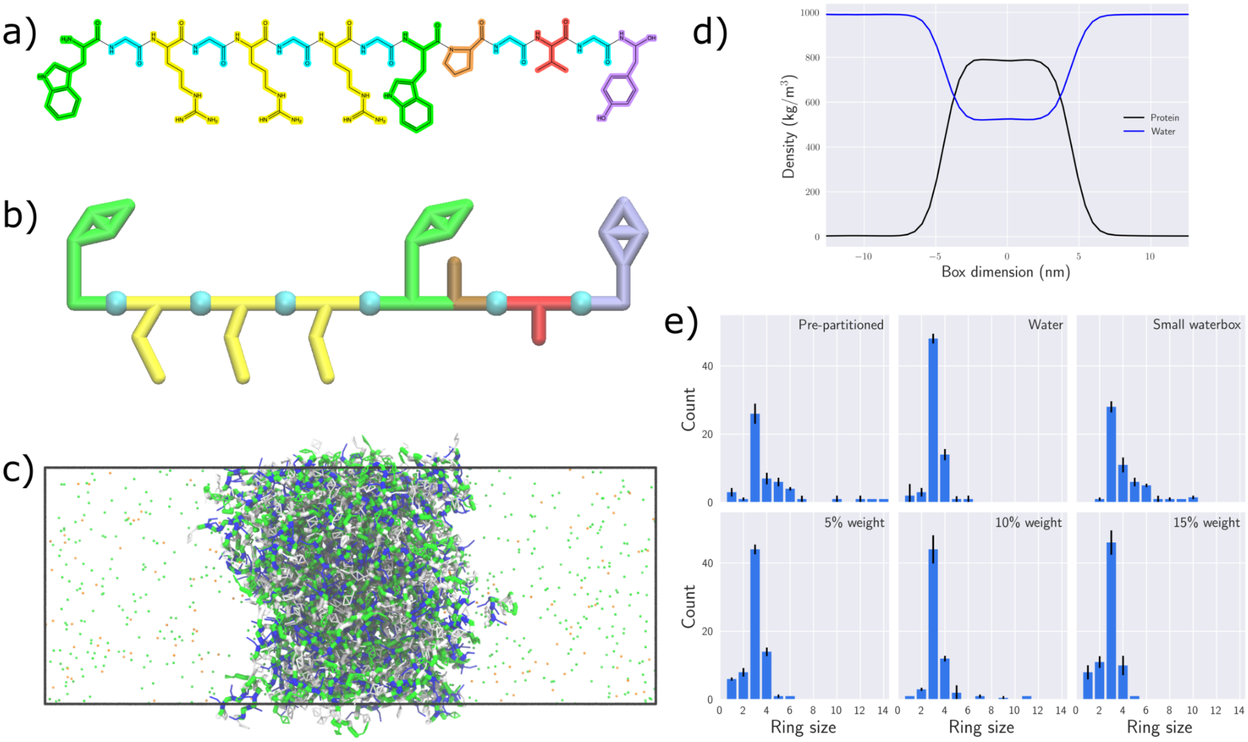

Figure S10. Simulations of WGR-1 peptide condensate systems. a) The chemical structure of the WGR-1 peptide. b) Martini coarse grained representation of the WGR-1 peptide. c) A condensate of WGR-1 peptides in a slab configuration. d) Density profiles of the water and protein components of the system in a slab configuration. e) Final ring size distributions in reactive Martini simulations.

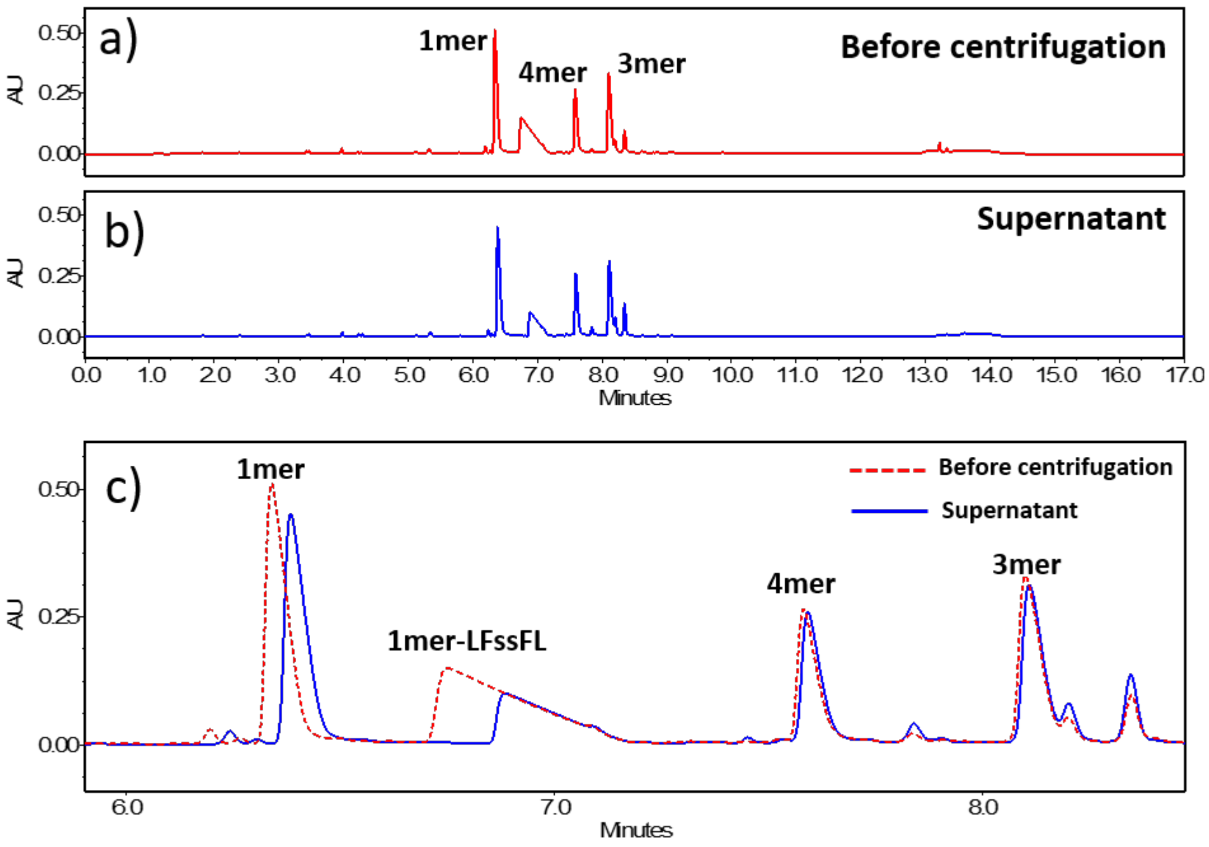

Figure S11. Partitioning experiment of 1mer in LFssFL condensates (a) UPLC chromatograms obtained from the condensate solution before centrifugation (b) UPLC chromatograms obtained from the supernatant solution after centrifugation (c) superimposed chromatograms of (a) and (b).

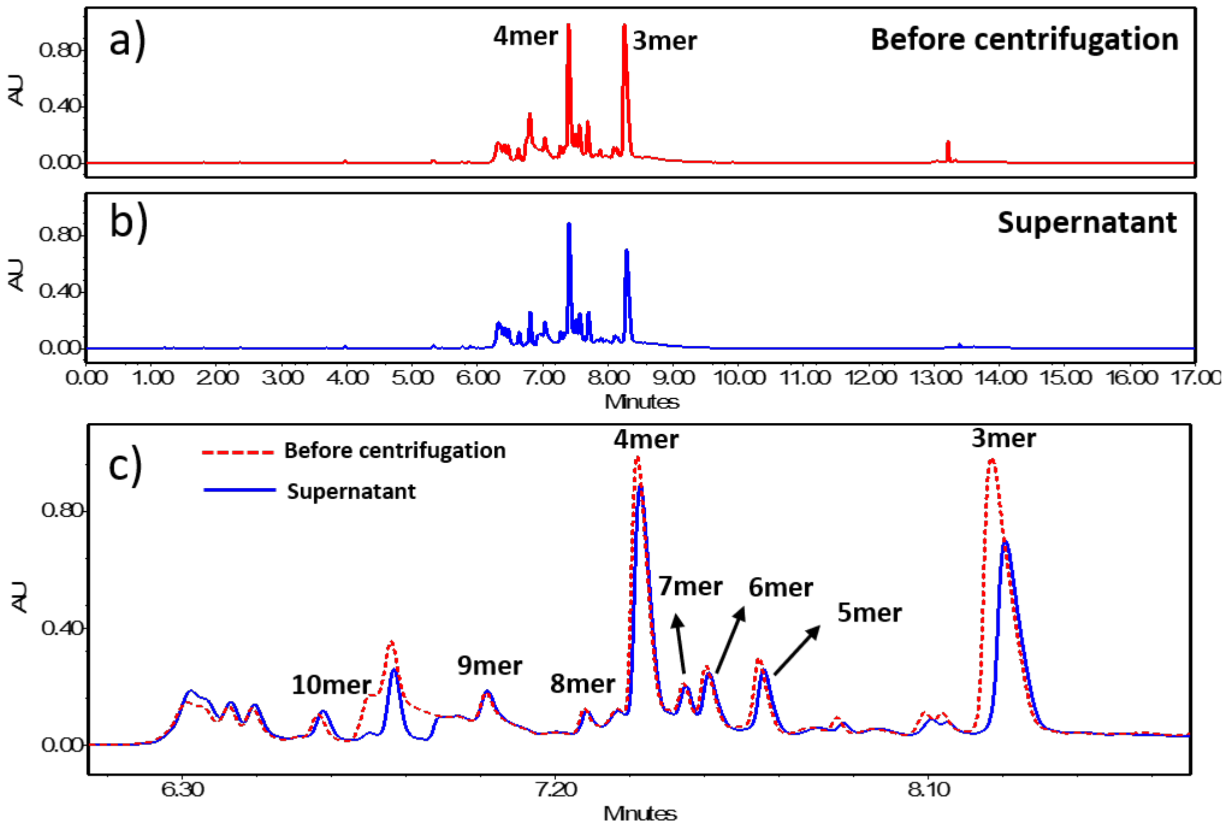

Figure S12. Partitioning experiment of 3mer and 4mer in LFssFL condensates (a) UPLC chromatograms obtained from the condensate solution before centrifugation (b) UPLC chromatograms obtained from the supernatant solution after centrifugation (c) superimposed chromatograms of (a) and (b).

	Partitioning (experimental)	Partitioning (simulation)
1mer	5.71 ± 0.65	55 ± 11
3mer	26.2 ± 1.3	n/a
4mer	6.99 ± 1.2	n/a
5mer	2.24 ± 1.2	n/a
6mer	2.26 ± 1.1	n/a
7mer	1.13 ± 0.1	n/a
8mer	1.6 ± 0.35	n/a
9mer	1.3 ± 0.2	n/a
10mer	1.7 ± 0.8	n/a

Table S5. Experimentally measured partition coefficients of unassembled macrocycles between the dense and dilute regions of LFssFL condensates.
